# Large-scale resting state network correlates of cognitive impairment in Parkinson's disease and related dopaminergic deficits

**DOI:** 10.3389/fnsys.2014.00045

**Published:** 2014-04-03

**Authors:** Alexander V. Lebedev, Eric Westman, Andrew Simmons, Aleksandra Lebedeva, Françoise J. Siepel, Joana B. Pereira, Dag Aarsland

**Affiliations:** ^1^Department of Psychiatry, Centre for Age-Related Medicine, Stavanger University HospitalStavanger, Norway; ^2^Department of Neurobiology, Care Sciences and Society, Karolinska InstitutetStockholm, Sweden; ^3^Department of Neuroimaging, Institute of Psychiatry, King's College LondonLondon, UK; ^4^NIHR Biomedical Research Centre for Mental Health and Biomedical Research Unit for DementiaLondon, UK

**Keywords:** parkinson's disease, cognition, dopamine, resting state fMRI, SPECT, graph theory, nodal strength, modularity

## Abstract

Cognitive impairment is a common non-motor feature of Parkinson's disease (PD). Understanding the neural mechanisms of this deficit is crucial for the development of efficient methods for treatment monitoring and augmentation of cognitive functions in PD patients. The current study aimed to investigate resting state fMRI correlates of cognitive impairment in PD from a large-scale network perspective, and to assess the impact of dopamine deficiency on these networks. Thirty PD patients with resting state fMRI were included from the Parkinson's Progression Marker Initiative (PPMI) database. Eighteen patients from this sample were also scanned with ^123^I-FP-CIT SPECT. A standardized neuropsychological battery was administered, evaluating verbal memory, visuospatial, and executive cognitive domains. Image preprocessing was performed using an SPM8-based workflow, obtaining time-series from 90 regions-of-interest (ROIs) defined from the AAL brain atlas. The Brain Connectivity Toolbox (BCT) was used to extract nodal strength from all ROIs, and modularity of the cognitive circuitry determined using the meta-analytical software Neurosynth. Brain-behavior covariance patterns between cognitive functions and nodal strength were estimated using Partial Least Squares. Extracted latent variable (LV) scores were matched with the performances in the three cognitive domains (memory, visuospatial, and executive) and striatal dopamine transporter binding ratios (SBR) using linear modeling. Finally, influence of nigrostriatal dopaminergic deficiency on the modularity of the “cognitive network” was analyzed. For the range of deficits studied, better executive performance was associated with increased dorsal fronto-parietal cortical processing and inhibited subcortical and primary sensory involvement. This profile was also characterized by a relative preservation of nigrostriatal dopaminergic function. The profile associated with better memory performance correlated with increased prefronto-limbic processing, and was not associated with presynaptic striatal dopamine uptake. SBR ratios were negatively correlated with modularity of the “cognitive network,” suggesting integrative effects of the preserved nigrostriatal dopamine system on this circuitry.

## Introduction

Cognitive impairment is a very important and common non-motor feature of Parkinson's disease (PD) with a major impact on patients' and caregivers' quality of life, as well as healthcare costs (Muslimovic et al., 2005; Vossius et al., 2011; Svenningsson et al., 2012). Approximately one-fifth of newly diagnosed PD patients fulfill clinical criteria for mild cognitive impairment (PD-MCI) (Aarsland et al., 2009) and about one-sixth develop dementia after 5 years (Williams-Gray et al., 2009).

Although the exact role and mechanisms of the dopaminergic system in cognition are still a matter of debate, there is no doubt that its preservation is crucial for cognitive functioning of PD patients. Thus, there is strong evidence suggesting that the impairment of at least 3 major dopaminergic pathways (nigrostriatal, mesocortical, mesolimbic) originating in the brainstem play a very important role in cognitive dysfunction associated with PD (Narayanan et al., 2013).

Previous neuroimaging studies assessing brain networks *in vivo* have shown impairment of the dopaminergic pathways and related neural circuits in PD. Numerous studies on cognitive dysfunction associated with PD have revealed structural and functional abnormalities within the cortico-strio-thalamo-cortical circuits, known to be largely modulated by the dopaminergic system (Hirano et al., 2012; Christopher and Strafella, 2013).

Decreased 6-[^18^F]-fluorodopa (^18^F-DOPA) uptake in the anterior cingulate cortex, ventral striatum and right caudate nucleus has been found in PD patients with dementia (PDD) compared to PD (Ito et al., 2002). Studies employing Single Photon Emission Computed Tomography (SPECT) with the dopamine transporter-binding ligands (DaTSCAN) also suggest more severe striatal presynaptic dopaminergic deficiency in PDD compared to PD patients, especially in the caudate nuclei (O'Brien et al., 2004). In addition, there is also evidence suggesting an association between striatal ^18^F-DOPA uptake and executive performance in PD patients (Bruck et al., 2001; Cheesman et al., 2005; Cropley et al., 2008).

Several ^18^F-fludeoxyglucose Positron Emission Tomography (FDG-PET) studies analyzing brain networks in PD have identified partially overlapping patterns of brain metabolic changes associated with cognitive impairment in multiple domains, suggesting that the PD-related profile of cognitive impairment is associated with reduced glucose metabolism mainly in prefrontal, parietal, hippocampal, and striatal regions (Mentis et al., 2002; Huang et al., 2007a,b; Eidelberg, 2009). H^15^_2_O-PET studies have shown an impaired basal ganglia and dorsolateral prefrontal response during executive task performance in PD (Owen et al., 1998; Dagher et al., 2001; Cools et al., 2002).

Functional MRI studies have also revealed abnormalities within the frontal-subcortical circuits in patients with PD. For instance, an abnormal fronto-striatal response during executive task performance has been found in cognitively impaired PD patients compared to non-impaired ones (Lewis et al., 2003). Another fMRI study assessing working memory and motor functions in ON and OFF dopaminergic medication states in PD patients (Mattay et al., 2002) found increased prefrontal and parietal activations during the working memory task performance in the OFF state, which were positively correlated with errors during the task. Studies focusing on set-shifting paradigms have found a PD-associated pattern of prefrontal and parietal response characterized by either reduced or increased activation depending on whether the caudate nucleus was involved in the task (Monchi et al., 2004, 2007).

Notably, a pharmacological fMRI study in healthy subjects revealed a significant effect of L-dopa administration on striatal functional connectivity (Kelly et al., 2009). In addition to its effects on motor networks, L-dopa increased functional connectivity between the ventral striatum and ventrolateral prefrontal cortex, and disrupted connectivity of the striatum with components of the default mode network (Kelly et al., 2009). Impaired deactivation of the default mode network during executive task performance has been reported in several fMRI studies of PD (Tinaz et al., 2008; Van Eimeren et al., 2009). Resting state fMRI studies have reported abnormal cortico-striatal connectivity in PD (Wu et al., 2009; Helmich et al., 2010; Kwak et al., 2010), while L-DOPA administration has been shown to enhance functional connectivity in the frontal areas of the sensorimotor network (Esposito et al., 2013).

The brain is a complex biological system that demonstrates emergent network properties on different scales, even at a cellular and single-structure level (Welsh et al., 2010). At the cellular scale, neocortical neurons are organized into sets of structurally and physiologically merged modules (Mountcastle, 1997), which in turn, are grouped into functionally segregated hypercolumns, wired with inter-modular connections. At the larger scale, system-wide coordination of the brain networks give rise to the coherent dynamic states that support cognitive functions and behavior (Sporns, 2013). Large-scale network architecture of the human brain appears to combine two principles of structural and functional organization. On the one hand, densely connected network modules or communities promote specialized processing and *functional segregation*. On the other hand, these specialized communities are interconnected via long-distance pathways that ensure efficient *functional integration* across multiple functional domains. Maintaining the balance between segregation and integration is thought to be essential for establishing complex network dynamics that support cognition (Sporns, 2010).

Recent advances in neuroscience and mathematical modeling have made it possible to apply classical concepts of graph theory to the analysis of brain network structure and dynamics (Rubinov and Sporns, 2010; Sporns, 2010). Graph-theoretical studies of structural and functional networks of the brain have revealed “small-world” properties (Achard and Bullmore, 2007), i.e., the coexistence of dense local connectivity with relatively sparse long-range connections. Such small-world networks combine high clustering with a relatively short path length between any pair of the elements (e.g., brain regions). The “small-world” model may be of functional importance as it balances functional segregation (high modularity or clustering) and functional integration (short path length) and thus offers a network architecture that may be well-suited for neuronal information processing (Sporns and Zwi, 2004).

To date, there are very few studies of PD employing graph theoretical framework for fMRI data analysis. Skidmore et al. found reduced whole-brain global efficiency in PD (Skidmore et al., 2011). Compared to healthy controls, 14 PD patients included in the study demonstrated reduced local efficiency (nodal level) in the precentral regions, primary and secondary visual cortex. Another recent study found global reduction of network-level processing efficacy in PD. Analysis of network modules indicated decreased interaction of the visual network with other brain modules, but abnormally increased connectivity within the sensorimotor network. The authors interpreted the latter as a compensatory mechanism aimed at overcoming the striato-cortical functional deficit within the motor loops, which may also be associated with loss of mutual inhibition between brain networks (Gottlich et al., 2013).

To the best of our knowledge, there are no previous studies assessing brain correlates of PD-related cognitive impairment employing both dopamine transporter imaging and fMRI with graph theory metrics.

In the present study, we assessed global and local network-level correlates of cognitive dysfunction and related dopaminergic impairment in PD using the graph theory metrics of nodal strength and modularity.

We hypothesized that the PD-related profile of cognitive impairment would be associated mainly with abnormalities within the fronto-subcortical (impaired cortico-striatal connectivity) and fronto-parietal circuits, which are closely related to nigrostriatal deficiency.

## Methods

The main workflow steps are illustrated in Figure S1.

### Inclusion and exclusion criteria

We included all 30 subjects (31 minus one subject excluded during quality control due to “cuts” of dorsal cortical areas) with rs-fMRI enrolled in the Parkinson's Progression Marker Initiative (PPMI) (in total 452 PD patients), a multicenter study launched in 2010 designed to identify progression biomarkers in newly diagnosed PD patients (www.ppmi-info.org/data).

*Inclusion criteria* required that subjects must have at least two of the following symptoms: resting tremor, bradykinesia, rigidity or either asymmetric resting tremor or asymmetric bradykinesia. In addition, the subjects had to be drug naïve, Hoehn and Yahr stage I or II at baseline, and a screening ^123^I-FP-CIT SPECT scan, sensitive to the loss of striatal dopamine transporter (DaT) binding.

*Exclusion criteria* were atypical PD syndromes due to drugs or metabolic disorders, encephalitis, or other degenerative diseases. In addition, it was required that the subject was not taking levodopa, DA agonists, MAO-B inhibitors, amantadine, or other PD medication; or had taken levodopa or dopamine agonists prior to baseline for more than a total of 60 days.

### Neuropsychological assessment

In addition to a cognitive screening test, the Montreal Cognitive Assessment (MoCA), all subjects underwent a neuropsychological test battery developed to assess major cognitive domains affected by PD.

Visuospatial function was evaluated using the 15-item version of the Benton's Judgment of Line Orientation Test, which examines the ability of a subject to estimate angular relationships between line segments by visually matching angled line pairs to 11 numbered radii forming a semi-circle (Benton et al., 1978).

Verbal memory was assessed using the Hopkins Verbal Learning Test-Revised (HVLT-R) (Shapiro et al., 1999), which consists of presenting a list of 12 words over three learning trials. With each repetition, subjects are expected to learn additional words on the list and increase their performance with each trial. Total immediate recall or encoding (sum of trial 1–3) and delayed recall (after 20–25 min) scores were included in this study.

Executive functions were evaluated using three semantic fluency tests (names of animals, fruits, and vegetables, in 1 min each), the MoCA subtests of phonemic fluency (words that start from the letter “F,” in 1 min) and alternating trail making (drawing a line, going from a number to a letter, in ascending order; score 0–1).

Attention was assessed by the Letter-Number Sequencing Test (LNST), in which a combination of numbers and letters is read to the subject who is then asked to recall the numbers, first in ascending order and then the letters in alphabetical order. The Symbol Digit Modalities Test (SDMT) was also used to assess attention, in which specific numbers had to be paired with geometric figures based on a reference key within 90 s.

### Cognitive domains

Three cognitive domains were calculated based on the standardized tests for memory, visuospatial, and attention/executive functioning. Raw values were converted to z-scores using the mean and standard deviation of the healthy control group. Domain composite scores were calculated by averaging z-scores of the standardized tests in each cognitive domain.

In the *memory domain*, three learning trials and the delayed recall of HVLT-R were included. The *visuospatial domain* included the Benton judgment of line orientation. The *attention/executive domain* included the LNST, SDMT, semantic fluency, and the phonemic fluency test. No corrections were performed to adjust the tests scores for age or gender given that the subsequent analyses included these variables as nuisances.

Since the calculated composite scores for cognitive domains were scaled and reflected positive cognitive performance (the higher the score, the better functioning in a corresponding domain), we defined the “motor” domain by inverting and scaling UPDRS-III raw scores in order to achieve the same variable scale and direction (higher scores correspond to better motor function) when assessing and plotting the results.

### Automated meta-analysis in neurosynth

In order to support our hypotheses and to objectively identify regions that are relevant for cognitive functions, an automated search using the meta-analytical software Neurosynth (http://neurosynth.org) was undertaken. This approach utilizes text-mining and machine-learning techniques to perform probabilistic mapping between neural and cognitive states (Yarkoni et al., 2011). In the present study, the Python-based version (https://github.com/neurosynth/neurosynth) was used. The database was accessed on 24.10.13, searching for the key-words “executive” (237 studies), “visuospatial” (*n* = 116) and “memory” (*n* = 1470).

After the search overlapping patterns were found between cognitive domains. They were in line with the regions that have revealed an association with cognitive impairment in PD highlighted in the introduction. Thus, the profile of visuospatial functions included prefrontal, parietal, and occipital regions. The “executive” pattern contained prefrontal [with more extended involvement of dorsolateral prefrontal cortex (DLPFC)], cingulate, superior parietal, temporo-occipital, basal ganglia, and cerebellar regions. Finally, the “memory” profile, in addition to prefrontal and parietal regions, also included hippocampus, temporal areas, and basal ganglia.

Due to the observed overlap, the resulting statistical maps were merged and overlaid with the Automated Anatomical Labeling (AAL) atlas in order to have an unbiased definition of ROIs associated with cognitive functions for further network analysis. The main steps of the meta-analysis and the resulting maps are illustrated in Figure S2.

### MRI

#### Image acquisition

A standardized MRI protocol included acquisition of whole-brain structural and functional scans on 3 Tesla Siemens Trio Tim MR system. More details can be found in the MRI technical operations manual at http://www.ppmi-info.org/.

3D T1 structural images were acquired in a sagittal orientation using a MPRAGE GRAPPA protocol with Repetition Time (*TR*) = 2300 ms, Echo Time (*TE*) = 2.98 ms, Field of View (*FoV*) = 256 mm, Flip Angle (*FA*) = 9° and 1 mm^3^ isotropic voxel.

For each subject, 212 BOLD echo-planar rs-fMRI images (40 slices each, ascending direction) were acquired during a 8 min, 29 s scanning session (acquisition parameters: *TR* = 2400 ms, *TE* = 25 ms, *FoV* = 222 mm, *FA* = 80° and 3.3 mm^3^ isotropic voxels). Subjects were instructed to rest quietly, keeping their eyes open and not to fall asleep.

### ^123^I-FP-CIT SPECT

In the fMRI + DaTSCAN subgroup (*n* = 18), only those PD patients who had both fMRI and DaTSCAN acquired within less than a week interval were included.

Image acquisition was performed 4 ± 0.5 h after injection of ^123^I-FP-CIT, a time-point at which striatal specific binding ratios are stable (Booij et al., 1999) with a target dose of 185 MBq. The radiopharmaceutical was provided as a unit dose and filled to a standard volume, which was re-assayed.

Raw projection data were acquired into a 128 × 128 matrix with steps of 3 or 4 degrees for the total projections. Image preprocessing (reconstruction, attenuation correction, spatial normalization) was performed using the Hermes software (Medical Solutions, Stockholm, Sweden) at a central SPECT Core lab in New Haven (Connecticut, United States). Specific binding ratios were calculated for the left and right caudate nuclei according to specific binding ratio = (L/R Caudate)/(Occipital area) − 1 and then averaged for further analysis.

### Image preprocessing

As a first step, a population template was generated from the bias-corrected T1 structural images using the Diffeomorphic Anatomical Registration Through Exponentiated Lie Algebra (DARTEL) algorithm (Ashburner, 2007) in order to improve normalization quality.

For the fMRI data, two initial echo-planar volumes were automatically removed by the scanner software to minimize T1 effects on the T2^*^ echo-planar images, and the remaining 210 volumes underwent preprocessing in the SPM8-based (http://www.fil.ion.ucl.ac.uk/spm) pipeline implemented in the Data Processing Assistant for Resting-State fMRI: Advanced Edition (DPARSFA, version 2.3) (Chao-Gan and Yu-Feng, 2010), installed within the MATLAB environment (Matlab 8.0 and Statistics Toolbox, 2012).

Next, functional images underwent the following preprocessing steps: spatial realignment and slice-timing correction, co-registration with the high-resolution structural scans. Finally, the co-registered BOLD volumes were normalized into standardized Montreal Neurological Institute (MNI) space using the DARTEL template and resampled to 3 mm^3^ isotropic voxels. Spurious variance was reduced by a voxel-specific head motion correction (Satterthwaite et al., 2013) and by regressing-out time-series from the white matter and cerebrospinal fluid. Next, the images were band-pass filtered to eliminate biologically non-relevant signals (Biswal et al., 1995; Lowe et al., 1998) (it was not necessary to use large smoothing kernels due to a ROI-based framework implemented in the study), and the resulting low-frequency fluctuations were extracted from 90 regions-of-interest (ROIs) defined in the AAL atlas (Tzourio-Mazoyer et al., 2002) and were used in the subsequent network analysis (Rubinov and Sporns, 2010).

### Data analysis

#### Network analysis

The data analysis workflow was developed in order to assess both regional and global network-level correlates of presynaptic DAT uptake and cognitive functions. To do this, two metrics were selected: *nodal strength* (local measure) and *modularity* of a network (global measure).

#### Generalization of nodal strength and modularity for positive and negative connections

In binarized networks, the number of edges emanating from a particular node is known as its degree. For non-binarized networks, this metric has generalization called nodal strength (weighted degree), defined as the sum of neighboring link weights (Rubinov and Sporns, 2010).

Although the source of negative correlations in rs-fMRI is still a matter of debate (Fox et al., 2009; Murphy et al., 2009), there is strong evidence supporting a biological origin (Chang and Glover, 2009). In light of this, generalizations of several weighted graph theory metrics have been developed taking into account negative correlations (Rubinov and Sporns, 2011).

Thus, nodal strength can be calculated for positive and negative connections. The corresponding definition is straightforward:

(1) $$S_{i}^{\pm }=\sum_{j\;\in \;N}w_{ij}^{\;\pm }$$

[Equation (1), adopted from Rubinov and Sporns, 2011] Where:

*N*—set of all nodes in the network;

(*i*, *j*)—link between nodes *i* and *j* (*i*, *j* ∈ *N*), associated with connection weights *w*_*ij*_ (0 < |*w*| < 1)

Nodal strength can therefore be computed for both positive and negative weights (±). In our analysis, the total strength of both positive and negative weights was used.

A widely used metric for network modularity is formally defined as the fraction of the edges that are within a given set of communities minus the expected fraction of edges if the network was randomly wired (Newman, 2006). The metric therefore serves as a global large-scale network measure that allows quantification of the community structure of the brain. Higher modularity values for a particular network are generally associated with denser within-modular connections, but sparser connections between nodes that are in different modules.

Generalization of the modularity to both positive and negative correlations is more complex than nodal strength due to the differences in significance of positive and negative weights when determining modularity-partitions. Therefore, a non-symmetric generalization of network modularity has been proposed as:

(2a) $$Q^{*}=Q^{+}+\frac{v^{-}}{v^{+}+\;v^{-}}Q^{-}$$

or more complete definition:

(2b) $$\begin{array}{l} Q^{*}=\frac{1}{v^{+}}\;\sum_{ij}\text{​}\;\left(w_{ij}^{+}-e_{ij}^{+}\right)\text{​}\delta_{M_{i}M_{j}}\\ \;-\frac{v^{-}}{v^{+}+v^{-}}\;\sum_{ij}\text{​}\;\left(w_{ij}^{-}-e_{ij}^{-}\right)\text{​}\delta_{M_{i}M_{j}} \end{array}$$

Both equations are adopted from Rubinov and Sporns (2011)

Where:

*Q*^±^—modularity;

(*w*^±^_*ij*_ − *e*^±^_*ij*_)—difference between present within-module connection weights *w* and chance-expected within-module connection weights *e*;

δ_*M*_*i*_*M*_*j*__ = 1, when *i* and *j* are in the same module and δ_*M*_*i*_*M*_*j*__ = 0 otherwise.

*v*^±^ = ∑_*i* ∈ *N*_*s*^±^_*i*_—total weight (the sum of all positive or negative weights)

The Brain Connectivity Toolbox (BCT, http://www.brain-connectivity-toolbox.net) (Rubinov and Sporns, 2010) was used to compute the described measures. Of note, connectivity matrices were neither thresholded nor binarized. Instead we employed a strategy that aimed to analyze weighted graphs by taking into account both positive and negative weights.

Next, the analysis proceeded in two directions with the aim of assessing local and global network-level correlates of cognitive functioning in PD and the impact of nigrostriatal dopaminergic deficiency on these networks.

All statistical analyses were performed using the R programming language, version 3.0.1 (R Core Team, 2013).

#### Dimensionality reduction: covariance patterns between nodal strength and cognitive functions

Partial Least Squares Regression (PLSR) was performed to reduce the dimensionality of the data, estimating latent components associated with composite scores for each domain (executive, memory, visuospatial).

PLSR is an effective data-driven method that allows high-dimensional associations between explanatory and response variables to be reduced into a small set of latent variables (LVs) (Wold et al., 1984). After decomposition, each of the LVs represents a distinct pattern of brain–behavior associations.

The following elements of these components were of particular interest in our study: (1) eigenvector (loadings) showing the degree to which a given LV contributes to the variance within the X-matrix (in our case, brain network measures), and (2) a set of scores representing a transform of a particular data-point into a latent component's space (the degree to which a given component is “represented” in a particular subject).

The models were assessed with leave-one-out cross-validation. As a result, 3 LVs minimizing total Root Mean Squared Error Prediction (RMSEP) for all 3 domains were selected. For details, see Figure S3. Individual LV scores were subsequently correlated with 3 cognitive domains using motor function, age, and sex as nuisance covariates.

GLM formula:

(3) $$\begin{array}{l} {\text{LV}}_{\text{N}}\text{–score}\sim (\text{executive domain})\;+\;(\text{memory domain})\\ \;+(\text{visuospatial domain})\;+\;(\text{motor domain})\\ \;+(\text{age})\;+\;(\text{sex}). \end{array}$$

Finally, the scores were correlated with mean caudate DaT binding ratios in order to investigate which of them were influenced by nigrostriatal dopamine deficiency. The analysis was focused only on the caudate nuclei (without putamen), as this striatal structure is well-documented to be involved in cognition.

Due to the concerns regarding potential influence of motion artifacts, in addition to a voxel-specific correction strategy (Satterthwaite et al., 2013), analysis of motion with respect to the variables of interest (cognitive, motor domains, and age) was also performed. For this purpose, first principal component extracted from the absolute mean displacement values (x, y, and z axes) as well as relative displacements were used.

None of our variables of interest (executive, memory, and visuospatial domains) demonstrated significant association. The only significant associations were found for the motor domain (*p*_mean displacement_ = 0.036; *p*_relative displacement_ = 0.035) and age (*p*_mean displacement_ = 0.026), as expected. These variables were included in the modes as nuisance covariates.

#### Impact of nigrostriatal deficiency on the modularity of cognitive brain circuitry

For the second part, adjacency matrices were constructed using 60 AAL ROIs identified during the meta-analysis step (see Meta-Analysis section and Figure S2). Next, modularity was estimated based on both negative and positive weights [as described in Equations 2a and 2b].

Finally, an association between network modularity and mean DaT uptake in the caudate nuclei was analyzed using linear modeling.

## Results

### Demographics and clinical data

Demographics and clinical characteristics are shown in Table 1.

**Table 1:** **Demographics and clinical data**.

	**Complete sample (***n*** = **30**)**		Subsample^*^ (***n*** = **18**)	
	**Mean [±SD]**	**Median (range)**	**Mean [±SD]**	**Median (range)**
Age	61.67 [±9.46]	62 (40–75)	60.11 [±9.04]	61 (44–75)
MoCA	26.67 [±3]	27 (15–30)	26.72 [±3.5]	27 (15–30)
ExecDom	−0.254 [±0.73]	−0.125 (−1.37–1.26)	−0.297 [±0.67]	−0.125 (−1.35–0.99)
MemDom	−0.51 [±1.2]	−0.41 (−2.81–1.47)	−0.73 [±1.26]	−0.51 (−2.81–1.25)
VspDom	0.06 [±0.78]	−0.06 (−1.57–0.95)	0.08 [±0.84]	0.44 (−1.57–0.95)
UPDRS III	20.2 [±10.6]	17 (7–47)	19.83 [±10.9]	17 (7–47)

Male/Female ratio was 2:1.

* Subsample of subjects who had both fMRI and DaTSCAN acquired within less than a week interval.

MoCA, the Montreal Cognitive Assessment; ExecDom, “Executive” domain; MemDom, “Memory” domain; VspDom, “Visuospatial” domain; UPDRS III, part III of the Unified Parkinson's Disease Rating Scale.

The data were representative of the entire DaTSCAN cohort of PD patients (results not shown). Of note, visuospatial functions were relatively less affected than executive and memory domains.

### Graph theoretical analysis

#### Brain-behavior covariance patterns

The analysis was performed with PLS LV-scores determined after the dimensionality reduction step (see corresponding Methods section).

#### Nodal strength

**Figure 1:**
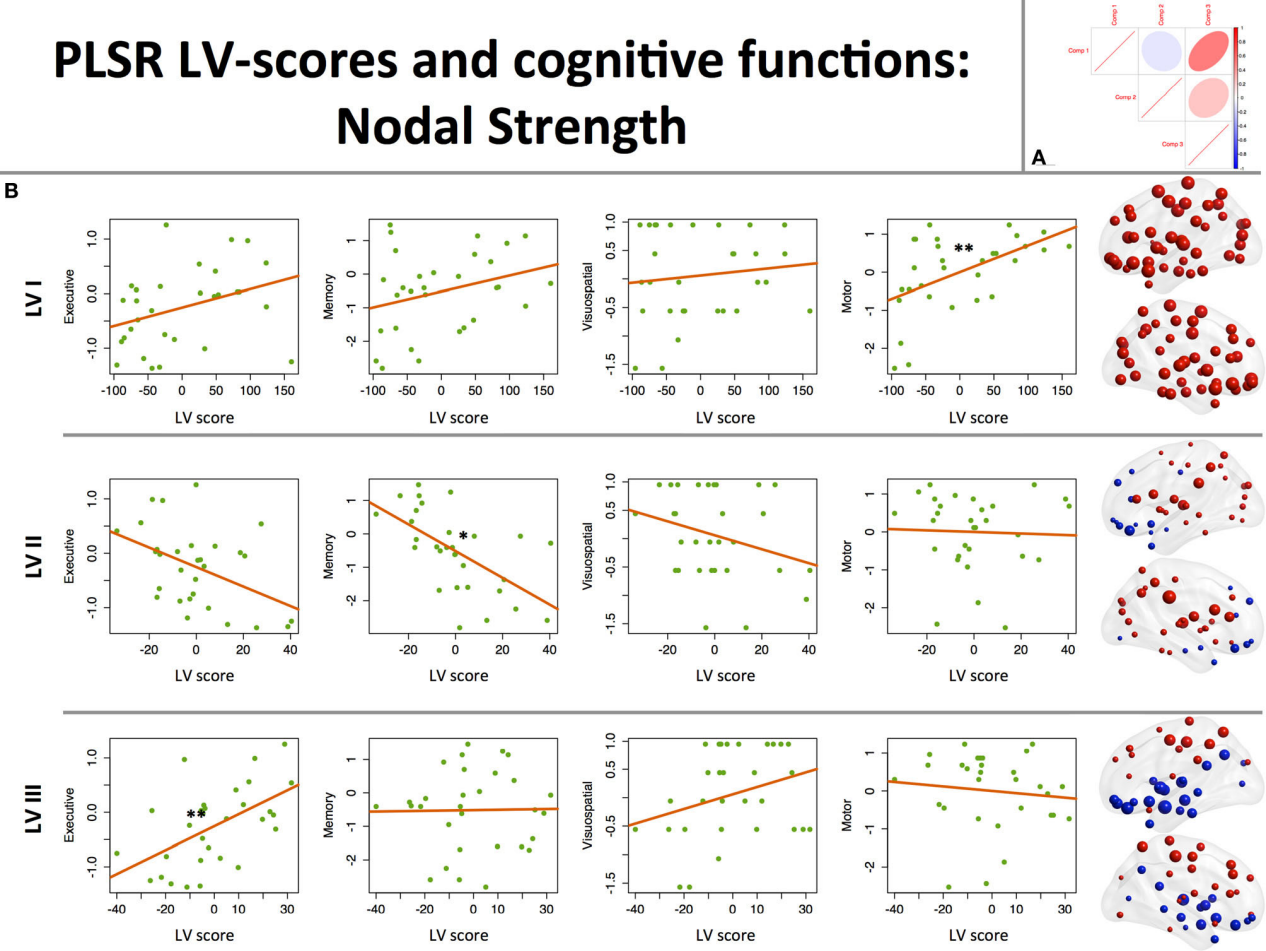
**Associations between component scores and behavioral data: nodal strength. (A)** Between-component correlation plot. Positive association (*r* = 0.5) was found between latent variables (LVs) I and III. **(B)** Associations between LV scores extracted from the nodal strength data and performance in 3 cognitive (executive, memory, visuospatial) and motor domains. On the right-hand side of the graph, corresponding loading maps are depicted in brain space, reflecting the relevance of the nodes (spheres) for a particular LV, the magnitude of which is represented by nodal size. Positive loading values are depicted as red spheres, whereas negative ones are shown in blue. ^*^p < 0.05, ^**^p < 0.01.

*The first PLS LV* captured global effects. Its higher scores were associated with higher strength of all 90 nodes with largest effects on motor, prefrontal cortices, and striatum. On a behavioral level, this component was positively associated with motor function (see Table 2, Figure 1).

**Table 2:**
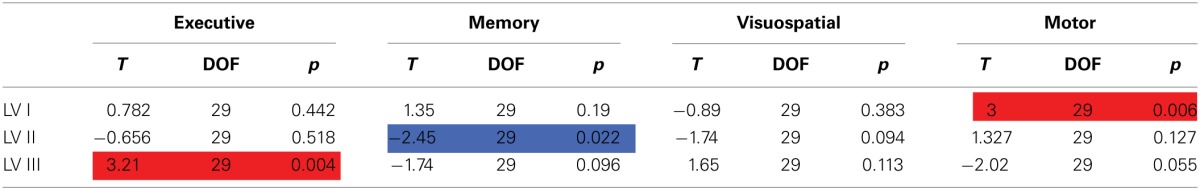
**Associations between component scores and behavioral data: nodal strength**.

The table shows associations between latent variable (LV) scores extracted from the nodal strength data and performance in 3 cognitive (executive, memory, visuospatial) and motor domains. Positive significant associations are depicted in red and negative ones in blue.

In total, 3 models were fitted with component scores as dependent variables, cognitive domains as independent variables (other covariates: motor domain score [also shown here as an important confounder], age and sex).

**T**, t-statistics; **DOF**, degrees of freedom.

*The second LV* was associated with higher degree of posterior (supramarginal, superior parietal, posterior cingulate, occipital regions) and striatal nodes, and lower prefronto-limbic (orbitofrontal, anterior cingulate, parahippocampal, temporopolar regions) nodal strength (except for operculo-triangular, middle frontal areas, and left hippocampus, which demonstrated positive associations). Behaviorally, this component displayed a negative association with memory function, that is to say that better memory performance was associated with reversed component pattern, favoring the involvement of prefronto-limbic nodes (see Table 2, Figure 1).

*The third LV*, in turn, favored cortical-subcortical segregation with positive associations found in dorsal cortical nodes (dorsolateral prefrontal, frontal and parietal areas) and negative in subcortical structures (hippocampi, striatum, globus pallidus), primary visual, middle temporal, and paralimbic (ventral prefrontal) areas. Higher scores of this component were associated with better executive performance (see Table 2, Figure 1).

#### Latent variable scores and caudate DaT uptake

**Figure 2:**
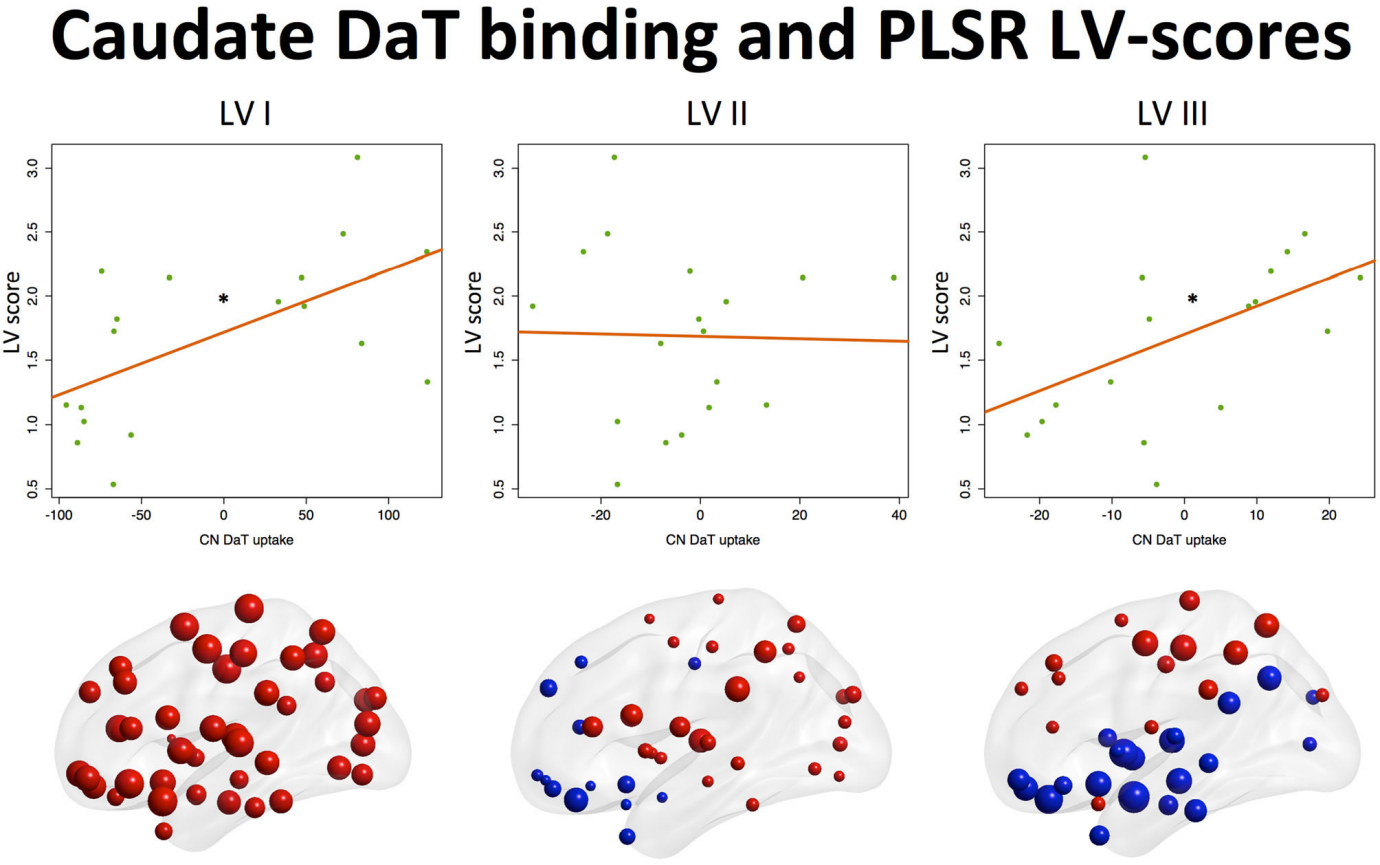
**Associations between component scores and mean caudate DaT binding**. Associations of latent variable (LV) scores extracted from the nodal strength data with nigrostriatal dopaminergic function measured by ^123^I-FP-CIT SPECT (mean caudate SBR ratios). Positive associations (^*^p < 0.05) were found for the Ist (“global/motor”) and IIIrd (“executive”) LVs. Corresponding loading maps are depicted in brain space, reflecting the relevance of the nodes (spheres) for a particular LV, the magnitude of which is represented by nodal size. Positive loading values are depicted as red spheres, whereas negative ones are shown in blue.

Analysis of the effects of nigrostriatal dopaminergic deficiency on the LVs estimated from the nodal strength revealed significant positive associations of mean caudate SBR ratios with I and III LV-scores (See Table 3, Figure 2).

**Table 3:**
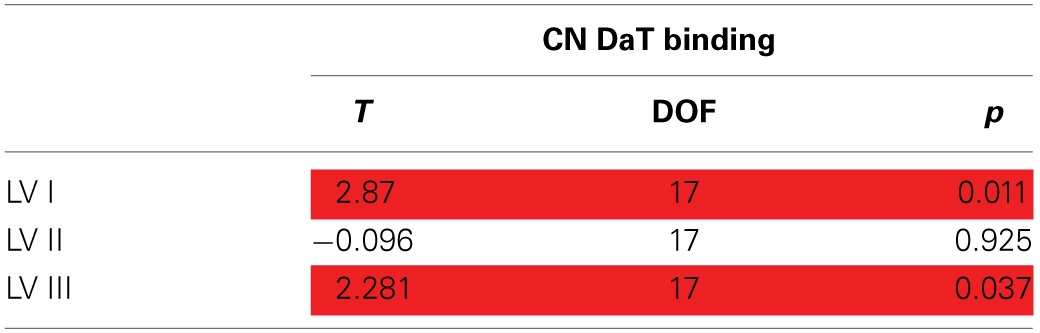
**Latent variable scores and mean caudate DaT binding**.

The table shows associations of the PLS latent variables (LVs) extracted during the dimensionality reduction step with nigrostriatal dopaminergic function measured by ^123^I-FP-CIT SPECT (mean caudate SBR ratios). Positive significant associations (depicted in red) were found for LVs I (“global/motor”) and III (“executive”).

CN, Caudate Nucleus; DaT, Dopamine Transporter; **T**, t-statistics; **DOF**, degrees of freedom.

This means that higher caudate DaT binding is associated with global increase of nodal strength and segregation toward more active dorsal cortical processing when the subject is at rest.

#### Modularity of the cognitive circuitry and caudate DaT binding

**Figure 3:**
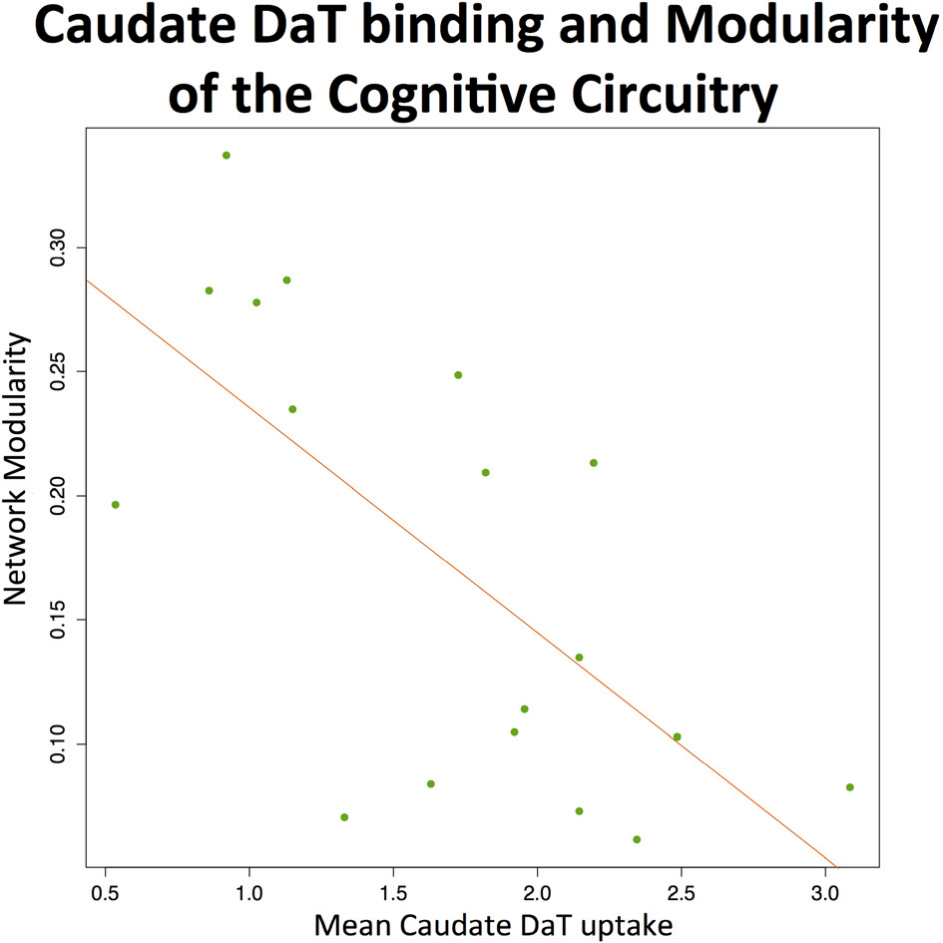
**Caudate DaT uptake and modularity of the cognitive circuitry at rest**. The figure shows significant (*p* < 0.01) negative association between modularity of the cognitive circuitry (identified with automated meta-analysis) and nigrostriatal dopaminergic function measured by ^123^I-FP-CIT SPECT (mean caudate SBR ratios).

The analysis revealed negative effects of the preserved dopaminergic function on modularity of the cognitive circuit (*T* = −3.6, 17 DOF, *p* = 0.002), suggesting greater integration among regions within this network (see Figure 3).

## Discussion

To the best of our knowledge, this is the first study to assess large-scale network correlates of PD-related cognitive impairment and presynaptic dopaminergic deficiency, combining rs-fMRI and DaTSCAN. Higher executive functional scores were associated with higher nodal strength of dorsal cortical nodes (predominantly in dorsolateral prefrontal, premotor, and superior parietal regions) and lower involvement of subcortical, occipital, temporal, and ventral cortical nodes, suggesting that relative preservation of executive functions in PD is linked to the dominance of dorsal cortical processing with inhibition of subcortical, paralimbic, and primary sensory circuitry when the subject is at resting state with eyes open. This pattern was positively influenced by higher nigrostriatal dopaminergic function.

Our results are consistent with an abnormally increased fronto-striatal connectivity found in a single-blind placebo-controlled rs-fMRI study of PD patients (Kwak et al., 2010), in which this hyperconnectivity was down-regulated by L-DOPA administration. Further analysis in this study revealed PD-related increase of power in the low-frequency band (0.02–0.05 Hz) in the striatum, which was also reduced after L-DOPA administration. Of note, this reduction correlated with L-DOPA-associated cognitive improvement. Apart from this, an increase in spontaneous oscillatory activity in the 10–35 Hz range (beta frequency band), occurring within the basal ganglia-thalamocortical networks and suppressed by dopaminergic treatment, is a well-replicated pathophysiological finding in PD (Brown et al., 2001; Levy et al., 2002; Gatev et al., 2006; Hammond et al., 2007), which provides additional support of our results converging from other imaging modalities.

The pattern associated with higher scores in the memory domain favoring prefronto-limbic processing did not reveal associations with presynaptic striatal dopamine uptake in the present study. The latter suggests that other mechanisms may be involved in the development of memory impairment associated with PD. The most likely ones are mesocortical dopaminergic deficiency (Narayanan et al., 2013) and impaired cortical cholinergic function (Bohnen et al., 2003), which in turn may at least be partly associated with concomitant cortical atrophy (Weintraub et al., 2012).

Of note, our study did not find any correlates of visuospatial impairment. However, this finding may be influenced by small sample size and due to the fact that visuospatial function was only mildly affected in the present cohort.

According to Mink's hypothesis (Mink, 1996), the basal ganglia play a crucial role in sustaining the balance between facilitation and suppression of movements. If we consider executive functions as the “movement of thoughts” a similar analogy can be drawn within this context. Indeed, cognitive frontal-subcortical loops is a widely accepted notion, where the DLPFC circuit has been documented to mediate set-shifting, complex problem-solving, retrieval abilities, organizational strategies, concept-formation, working memory (Zgaljardic et al., 2006), and other executive functions that are known to be affected in PD. Preserved nigrostriatal dopamine function therefore not only allows effective execution and termination of motor activity, but may also implement smooth switching between cognitive patterns, controlling mutual inhibition and/or facilitation of fronto-subcortical circuits. This is also supported by computational models of the basal ganglia that highlight their routing role in various cognitive functions, such as for example action-selection (Stocco et al., 2010).

In general, higher DaT binding values were associated with global integrative effects on the brain (global increase of nodal strength). This was also confirmed for the cognitive circuitry (defined during meta-analysis), where higher DaT SBR ratios (relative preservation of dopaminergic function) were associated with lower network-level modularity, suggesting that dopamine favors integration of the cognitive network when the subject is at rest. These results are in line with previous fMRI studies that indicated globally impaired network-level processing in PD (Skidmore et al., 2011; Gottlich et al., 2013). Negative effects of the preserved dopaminergic function on the modularity of cognitive circuitry are also in line with previous literature. Thus, a recent randomized double-blind rs-fMRI study of healthy subjects with bromocriptine administration (dopamine agonist) revealed drug-induced decreases in modularity, estimated for the whole brain (White et al., 2013). Our results also suggest that preserved nigrostriatal dopaminergic system allows supporting integrity of the cognitive network when a subject is at rest. In the light of this, it would be interesting to assess the dynamics of cognitive circuitry during performance of particular executive tasks or multi-tasking, where dopamine may have different or even opposite effects on network modularity. This is supported by previous functional imaging studies of executive functions in PD, revealing that hypodopaminergic states are associated with increased prefrontal cortical responses during performance of corresponding tasks (Mattay et al., 2002), whereas L-dopa administration, in contrast, decreases it (Cools et al., 2002). In this context, it is also worth mentioning an event-related fMRI study that found PD-related brain abnormalities during performance of the set-shifting task specifically developed to elicit caudate responses (Monchi et al., 2007). Compared to the control group, patients demonstrated increased cortical activation in the condition not specifically requiring the caudate nucleus, whereas decreased cortical activation was observed in the task that involved the caudate nucleus. These studies, however, are not focused on any specific dopaminergic system, looking at general dopamine-related effects instead, and therefore do not necessarily confirm the role of exactly nigrostriatal dopaminergic system in these phenomena.

Finally, a recently published graph theoretical MEG study with longitudinal design found that progression of PD is associated with growing impairment of local integration (measured by clustering coefficient) in multiple frequency bands and loss of global brain network efficiency (based on path length) in the alpha2 frequency band. This deterioration was, in turn, correlated with cognitive and motor impairment observed during disease progression (Olde Dubbelink et al., 2014). These findings provide additional support for our results, also suggesting global positive effects of dopaminergic preservation on efficient functioning of the brain networks.

The main limitation of the present study is a relatively small sample size. In addition, the cross-sectional design complicates causal interpretation of the results. Apart from this, the resting state setting itself hampers direct interpretation of the findings with regard to the role of brain networks in cognitive task performance. Active cognitive processing is likely associated with patterns of brain dynamics that are different compared to the ones occurring when the subject is at rest. These patterns in turn may have different associations with altered dopaminergic function in PD. Therefore, this presents a need for further studies of brain dynamics underlying cognitive processing in PD and related dopaminergic deficits.

The main strengths are a multimodal approach and graph theoretical setting that have not yet been implemented together for clarifying brain mechanisms of PD-related cognitive impairment. Further strengths are a relatively broad cognitive evaluation combining multiple tests to assess three major functional domains and the drug-naïve status of the participants. Of note, although the present study specifically investigated the nigrostriatal system, the deficiency measured by ^123^I-FP-CIT SPECT might also reflect indirect effects of neurodegeneration of dopaminergic neurons within other pathways, since the severity of dopaminergic deficits may correlate across different systems. Therefore, the results should be interpreted with caution.

To summarize, our study found that PD-related executive impairment is associated with altered balance between cortical and subcortical processing at rest, when contribution of the dorsal cortex is getting abnormally suppressed, and subcortical processing is disinhibited. This pattern (unlike brain profiles of visuospatial and memory impairment) is linked to nigrostriatal deficiency, which also has disruptive effects on cognitive circuitry at the network scale.

The results provide evidence for the contribution of the nigrostrital dopaminergic system in human cognition, and the described concept can potentially be utilized in future interventional studies to monitor the effects of treatments, including the approaches that augment cognitive functions.

### Conflict of interest statement

The authors declare that the research was conducted in the absence of any commercial or financial relationships that could be construed as a potential conflict of interest.

## Supplementary material

Figure S1 **Study Workflow.** The study workflow consisted of IV main steps: (I) Selection of 30 PD subjects with fMRI data from the complete PPMI cohort and an fMRI + DaTSCAN subsample of 18 subjects; (II) Preparation of the data for further analysis that, in turn, included (IIa) calculation of composite scores for 3 cognitive domains, (IIb) automated meta-analysis to define “cognitive network,” (IIc) image preprocessing and network measure extraction; (III) Dimensionality reduction with PLS followed by parametric tests evaluating associations between latent variable (LV) scores and cognitive functions; (IV) Final analysis assessing influence of caudate dopamine transporter (DaT) uptake on LV scores and modularity of the “cognitive network.

Figure S2 **Automated meta-analysis workflow.** An automated search using the meta-analytical software Neurosynth (http://neurosynth.org) was undertaken in order to identify regions that are relevant for cognitive functions. The key-words “executive” (237 studies), “visuospatial” (*n* = 116) and “memory” (*n* = 1470). The profile of visuospatial functions included prefrontal, parietal, and occipital regions. The “executive” pattern contained prefrontal (with more extended involvement of DLPFC), cingulate, superior parietal, temporo-occipital, basal ganglia, and cerebellar regions. Finally, the “memory” profile, in addition to prefrontal and parietal regions, also included hippocampus, temporal areas, and basal ganglia. Due to the observed overlap, the resulting statistical maps were merged and overlaid with the Automated Anatomical Labeling (AAL) atlas defining cognitive circuitry, the modularity of which was then correlated with nigrostriatal function measured by ^123^I-FP-CIT Single-Photon Emission Computed Tomography.

Figure S3 **Latent variable selection.** The figure shows Root Mean Squared Error Prediction (RMSEP) as a function of a number of PLS latent variables (LVs). The maximum number of LVs was selected that minimized total training (red dashed line) and leave-one-out cross-validation (green solid line) errors for all the domains (*n* = 3).
