# Supplementary material for: Large-scale resting state network correlates of cognitive impairment in Parkinson's disease and related dopaminergic deficits
Source: Front Syst Neurosci. 2014 Apr 3;8:45. doi: 10.3389/fnsys.2014.00045 (PMC3982053; doi:10.3389/fnsys.2014.00045)
Supplement: Figure S1 — Study Workflow. The study workflow consisted of IV main steps: (I) Selection of 30 PD subjects with fMRI data from the complete PPMI cohort and an fMRI + DaTSCAN subsample of 18 subjects; (II) Preparation of the data for further analysis that, in turn, included (IIa) calculation of composite scores for 3 cognitive domains, (IIb) automated meta-analysis to define “cognitive network,” (IIc) image preprocessing and network measure extraction; (III) Dimensionality reduction with PLS followed by parametric tests evaluating associations between latent variable (LV) scores and cognitive functions; (IV) Final analysis assessing influence of caudate dopamine transporter (DaT) uptake on LV scores and modularity of the “cognitive network. [file Presentation1.PDF]

SUPPLEMENTARY MATERIAL

Figure A1. Study Workflow.

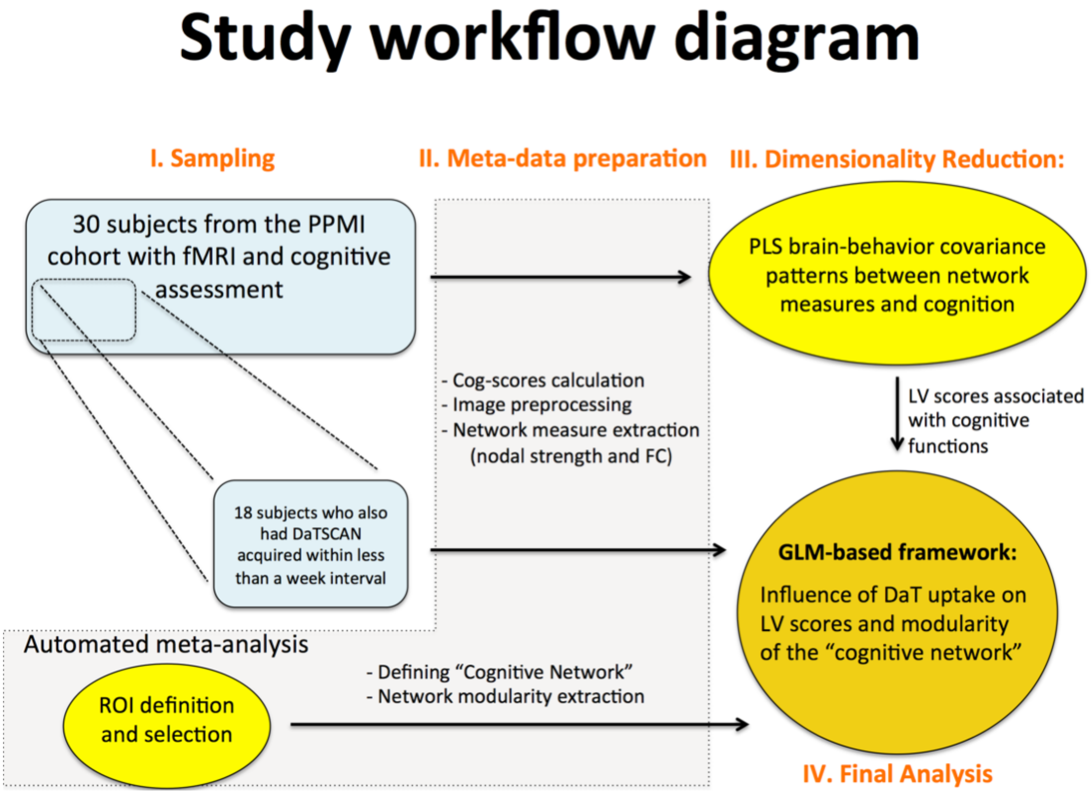

The study workflow consisted of IV main steps:
(I) Selection of 30 PD subjects with fMRI data from the complete PPMI cohort and an fMRI+DaTSCAN subsample of 18 subjects;
(II) Preparation of the data for further analysis that, in turn, included (IIa) calculation of composite scores for 3 cognitive domains, (IIb) automated meta-analysis to define "cognitive network", (IIc) image preprocessing and network measure extraction;
(III) Dimensionality reduction with PLS followed by parametric tests evaluating associations between latent variable (LV) scores and cognitive functions;
(IV) Final analysis assessing influence of caudate dopamine transporter (DaT) uptake on LV scores and modularity of the "cognitive network".

Figure A2. Automated meta-analysis workflow.

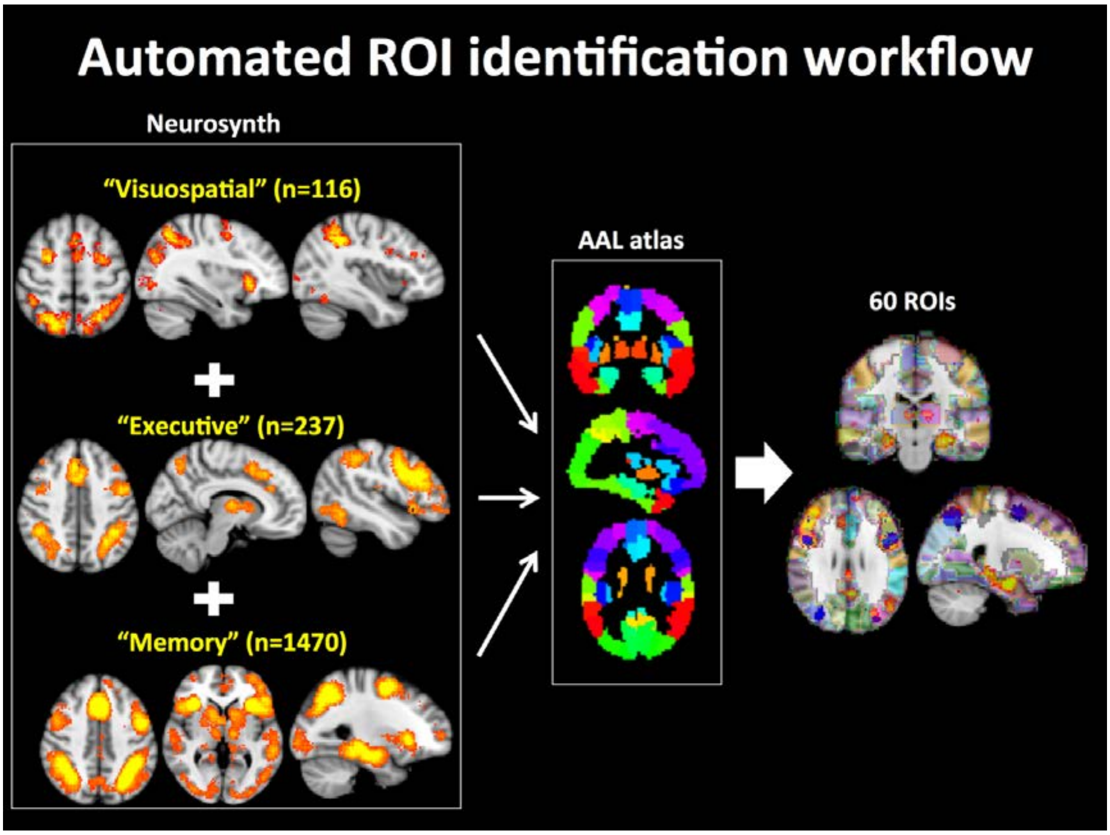

An automated search using the meta-analytical software Neurosynth (http://neurosynth.org) was undertaken in order to identify regions that are relevant for cognitive functions. The key-words "executive" (237 studies), "visuospatial" (n=116) and "memory" (n=1470).

The profile of visuospatial functions included prefrontal, parietal and occipital regions. The "executive" pattern contained prefrontal (with more extended involvement of DLPFC), cingulate, superior parietal, temporo-occipital, basal ganglia and cerebellar regions. Finally, the "memory" profile, in addition to prefrontal and parietal regions, also included hippocampus, temporal areas and basal ganglia.

Due to the observed overlap, the resulting statistical maps were merged and overlaid with the Automated Anatomical Labeling (AAL) atlas defining cognitive circuitry, the modularity of which was then correlated with nigrostriatal function measured by ^{123}I-FP-CIT Single-Photon Emission Computed Tomography.

Figure A3. Latent variable selection

PLSR model cross-validation

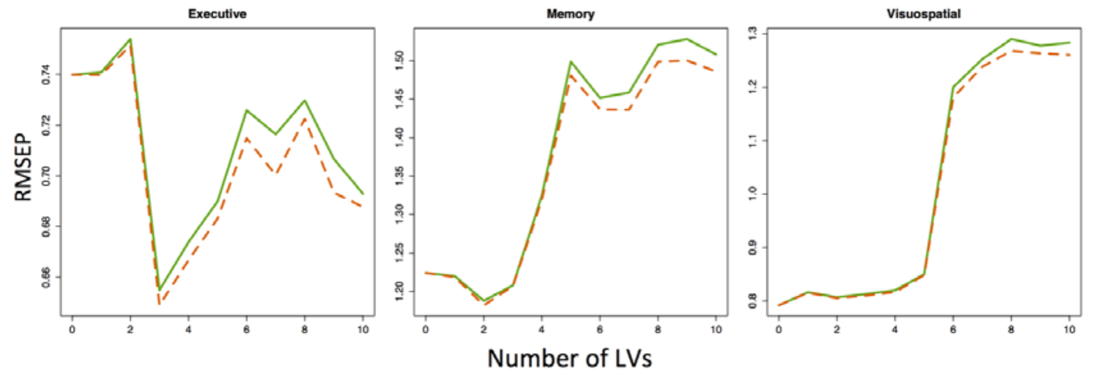

The figure shows Root Mean Squared Error Prediction (RMSEP) as a function of a number of PLS latent variables (LVs). The maximum number of LVs was selected that minimized total training (red dashed line) and leave-one-out cross-validation (green solid line) errors for all the domains (n=3).
